# Determinants of Heterogeneous Blood Feeding Patterns by *Aedes aegypti* in Iquitos, Peru

**DOI:** 10.1371/journal.pntd.0002702

**Published:** 2014-02-13

**Authors:** Kelly A. Liebman, Steven T. Stoddard, Robert C. Reiner, T. Alex Perkins, Helvio Astete, Moises Sihuincha, Eric S. Halsey, Tadeusz J. Kochel, Amy C. Morrison, Thomas W. Scott

**Affiliations:** 1 University of California, Davis, Davis, California, United States of America; 2 Fogarty International Center, National Institutes of Health, Bethesda, Maryland, United States of America; 3 Naval Medical Research Unit No. 6, Iquitos, Peru; 4 Hospital Apoyo, Iquitos, Peru; 5 Naval Medical Research Unit No. 6, Lima, Peru; 6 Naval Medical Research Center, Silver Spring, Maryland, United States of America; Centers for Disease Control and Prevention, United States of America

## Abstract

**Background:**

Heterogeneous mosquito biting results in different individuals in a population receiving an uneven number of bites. This is a feature of many vector-borne disease systems that, if understood, could guide preventative control efforts toward individuals who are expected to contribute most to pathogen transmission. We aimed to characterize factors determining biting patterns of *Aedes aegypti*, the principal mosquito vector of dengue virus.

**Methodology/Principal Findings:**

Engorged female *Ae. aegypti* and human cheek swabs were collected from 19 houses in Iquitos, Peru. We recorded the body size, age, and sex of 275 consenting residents. Movement in and out of the house over a week (time in house) and mosquito abundance were recorded on eight separate occasions in each household over twelve months. We identified the individuals bitten by 96 engorged mosquitoes over this period by amplifying specific human microsatellite markers in mosquito blood meals and human cheek swabs. Using a multinomial model assuming a saturating relationship (power), we found that, relative to other residents of a home, an individual's likelihood of being bitten in the home was directly proportional to time spent in their home and body surface area (*p*<0.05). A linear function fit the relationship equally well (ΔAIC<1).

**Conclusions/Significance:**

Our results indicate that larger people and those who spend more time at home are more likely to receive *Ae. aegypti* bites in their homes than other household residents. These findings are consistent with the idea that measurable characteristics of individuals can inform predictions of the extent to which different people will be bitten. This has implications for an improved understanding of heterogeneity in different people's contributions to pathogen transmission, and enhanced interventions that include the people and places that contribute most to pathogen amplification and spread.

## Author Summary

We studied the biting habits of *Aedes aegypti*, the principal vector of dengue virus, to determine why certain people are bitten more often by this day-active mosquito. Over one year in dengue-endemic Iquitos, Peru, we collected blood fed mosquitoes from 19 households. Mosquito blood meals were then matched to household residents using genetic fingerprinting. We found that within a household, larger individuals and those spending more time in the home were bitten more often than other household residents. Importantly, our results show that one's probability of being bitten is dependent on the characteristics of other household residents and visitors. These results indicate that measurable characteristics of individuals do predict who is most exposed to mosquito-borne pathogens, which contributes to our understanding of pathogen transmission processes, informs development of mathematical disease models, and can enhance the design of targeted control programs.

## Introduction

Mosquito blood feeding behavior is epidemiologically important because of its central role in determining which vertebrate hosts and mosquitoes are exposed to a pathogen. *Aedes aegypti*, the principal mosquito vector of dengue (DENV) and urban yellow fever viruses [1] is highly anthropophilic, feeding predominantly on people during daylight hours and tending to travel short distances to obtain its blood meals [2], [3], [4], [5]. Females often take more than one blood meal per gonotrophic cycle [6], increasing their probability of (1) imbibing an infected blood meal and (2) after surviving an extrinsic incubation period, becoming infectious, and transmitting virus to an uninfected person [7]. These behaviors lead to the assumption that the risk of DENV infection is highest at the scale of individual locations; the places where female *Ae. aegypti* feed and people live or visit [8], [9], [10], [11], [12]. Even at this fine scale, however, predicting infection risk remains difficult because some individuals are bitten more often than others for reasons that are poorly understood [13], [14], [15], [16], [17], [18], [19], [20].

A better understanding of who gets bitten more often and why would be useful for designing targeted methods of dengue prevention as well as for developing mathematical models of virus transmission. Although models have traditionally assumed that mosquitoes bite people randomly [21], growing empirical evidence indicates that mosquito biting patterns are heterogeneous and theoretical work indicates that this can have important impacts on transmission dynamics [22], [23], [24]. In particular, people who receive many more mosquito bites than others could act as superspreaders of a pathogen, infecting a disproportionate number of vectors and thus playing a central role in pathogen transmission dynamics [25]. Identifying these people is, therefore, key for effective, targeted disease control strategies [20]. A number of factors have been identified that may make some people more likely to be bitten than others: host body size (larger people being bitten more often), infection with parasites, body temperature, age (perhaps as a proxy for other biological factors), sex, semiochemicals, microflora on the skin, and host movement and defensive behavior [10], [12], [14], [16], [18], [19], [26], [27], [28], [29]. In the case of *Ae. aegypti*, results from a study conducted in Puerto Rico indicated that people under 20 years of age received fewer bites than those 20 years and older, regardless of gender [15]. There are several plausible explanations for the detected differences, including variation in individual body size and host movement patterns [10].

Our understanding of why some hosts are bitten more often by *Ae. aegypti* is incomplete, in part, because most studies do not account for the many potentially important differences among human hosts that could influence the chance of receiving a mosquito bite. Variation in biting patterns could be due to differences in inherent attractiveness to mosquitoes, determined by body size or smell, or some other characteristic that has yet to be identified. Observed variation in biting could also be due to the amount of time an individual spends in the same house as biting mosquitoes. We suspect that the most likely explanation combines individual characteristics and exposure time as principal determinants governing which individuals mosquitoes tend to bite most often. For instance, children may receive fewer bites than adults because they are smaller, exposed to fewer mosquitoes during the day or more active than adults. In Iquitos, Peru, for instance, mosquito abundances were found to be very low in schools compared to households [30]. During major portions of the day, when they are at school, children in Iquitos may be physically removed from biting mosquitoes. It is also important to consider the other individuals available at a particular location for mosquitoes to bite. Although mosquitoes may find a given individual suitable for biting, he or she may not be bitten if there are other people in the home that spend more time there or are more attractive to biting mosquitoes. Likewise, if mosquitoes only ever encounter a single individual, they will likely bite that person regardless of how attractive or unattractive they are. Making inferences about the factors that contribute to one's risk of being bitten requires simultaneously accounting for the characteristics of other potential blood meal hosts in the locations where mosquito encounters take place.

In this study, we sought to isolate individual-level factors driving *Ae. aegypti* biting patterns by identifying which people living in 19 houses in Iquitos, Peru were bitten most often over a 12-month period. The person bitten was determined by DNA profiling of blood in engorged mosquitoes collected inside each house. We then assessed how a number of factors affected each participant's probability of receiving a bite. Our analysis revealed that some individuals are indeed bitten more often than others and that human exposure time and body surface area are associated factors with this heterogeneity.

## Methods

### Data

#### Ethics statement

All participants in this study provided oral informed consent, which was recorded by investigators before the first interview was conducted. For minors, informed consent was provided by a parent or guardian. Written consent was not taken/required for this protocol because the study was considered minimal risk and non-invasive by reviewing bodies. The protocol for this study was approved by the University of California, Davis (Protocol #2007-15244) and the U.S. Naval Medical Research Detachment (currently the U.S. Naval Medical Research Unit No. 6, Protocol #NMRCD. 2007.0007). Both institutional review boards were in compliance with all federal regulations governing the protection of human subjects, and the latter was also registered with Peruvian network of ethics committees to ensure compliance with Peruvian regulations.

#### Participants

**Figure 1:**
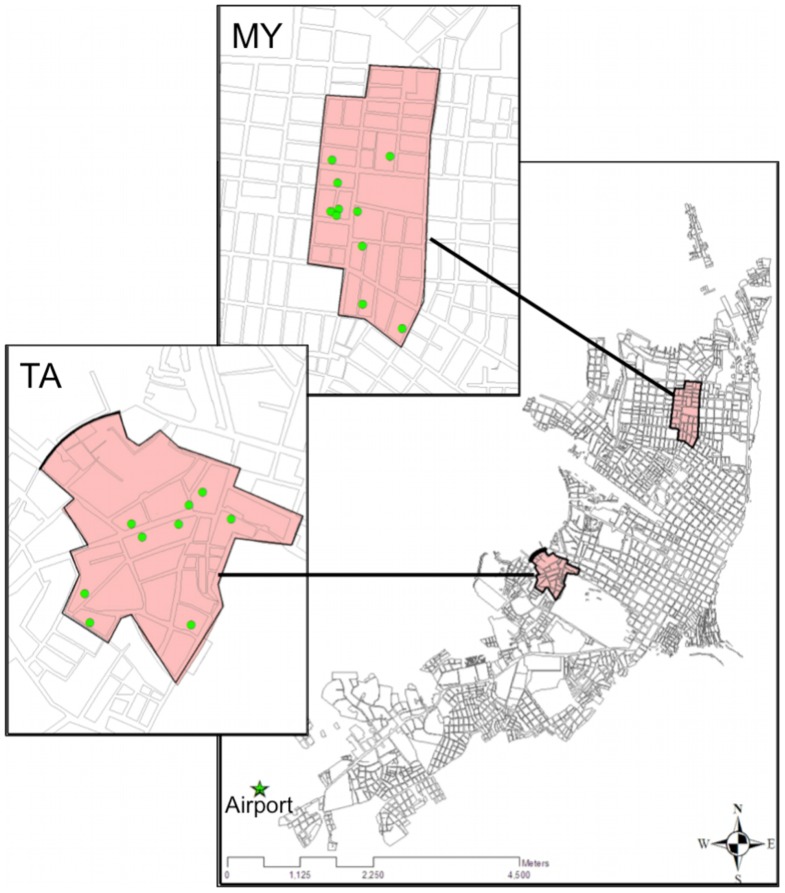
Houses of study participants (denoted by green dots) were chosen from two neighborhoods in Iquitos, Maynas (MY) and Tupac Amaru (TA).

Study houses were identified from a larger group of households under both active and passive surveillance for DENV in Maynas (MY) and Tupac Amaru (TA); two Iquitos neighborhoods (Figure 1) [8], [31], [32]. Selected homes contained a minimum of eight permanent residents at the initiation of the study during 2009, with more than 90% willing to provide cheek swab samples and at least one activity interview. A description of house construction in the study neighborhoods is provided by Getis et al [8]; windows were not screened, and bed nets (insecticide treated or untreated) were not used by the study population.

#### Interviews and mosquito collections

To obtain participant anthropomorphic measurements and time spent in house information, interviews were conducted in each house at 4–8 week intervals. Between October 2009 and June 2010, interviews were conducted bimonthly. From June–October 2010 the frequency of interviews was increased to once per month. During each interview period *k*, for each participant *i*, we recorded age (denoted *A_i|k_* and sex (denoted *G_i_*), and obtained height (denoted *H_i|k_*) and weight (denoted *W_i|k_*) measurements, to be used as independent variables for statistical analysis. The body surface area (m^2^) (*S_i|k_*) of an individual *i* was calculated for each interview period *k* using the Dubois equation [33], as described by Port et al. [18]:
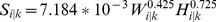
(1)where *W_i|k_* is weight in kilograms and *H_i|k_* is height in meters. To determine the amount of time, *T_i|j,k_*, that each resident *i* of house *j* spent at home during the interview week *k*, participants were asked what hours they intended to be home, between 05:00 and 22:00, from Saturday through Friday of that week. Interviews were typically conducted on Saturday, prospectively obtaining daily activity information for each individual from that day forward. Some individuals were interviewed later in the week, depending on their schedules (Figure S1).

Mosquitoes were collected concurrently from each house during interview weeks. Adult mosquitoes were removed twice daily (morning and afternoon) Monday–Friday using Prokopack aspirators [34]. Adult mosquito collections were attempted from all rooms in each study house. The time spent collecting varied depending on the size of the house and the number of mosquitoes encountered. On average, aspiration collections lasted 10–25 minutes.

#### Sample collection and processing

On the date of enrollment, a cheek swab was taken from each participant to determine that individual's unique DNA profile. Samples were collected by rubbing the inner surfaces of the participants' cheeks with wooden applicator sticks and suspending cells in 400 uL lysis buffer (1% SDS, 50 mM EDTA, 10 mM TRIS-HCL), which consistently yields 2–6 ng of human DNA [35]. Vials were labeled with the participant's identifying number and house code, evenly aliquoted into two labeled vials, and stored at −80°C until DNA extraction. DNA was extracted from each cheek swab using a modified version of the Qiagen QIAamp DNA Micro kit (Qiagen). Briefly, 200 uL of cells suspended in lysis buffer were added to the supplied columns and spun for 30 seconds, followed by washes with 500 uL of AW1 and AW2 buffers provided in the Qiagen kit. Samples were then eluted in 25 uL of the provided Qiagen buffer and stored at −80°C until analysis.

After each mosquito collection, adult mosquitoes were sedated at −20°C and identified to species. *Ae. aegypti* males and females were stored individually in 0.65 ml microcentrifuge tubes and labeled with the date, time, and collection house number and transferred to −80°C for storage. From female mosquitoes we removed and stored (in −80°C) legs, head/throax, and abdomens in separate vials. Abdomens were classified based upon the amount of blood they contained by external examination: engorged, partially engorged, or not engorged [15].

Engorged and partially engorged abdomens were suspended and ground in 175 uL of RPMI medium. DNA was extracted from blood in mosquito midguts collected between October 2009 and October 2010 using the Qiagen AllPrep DNA/RNA Mini Kit following manufacturer protocols. Extracted DNA was re-suspended in 25 uL of the supplied elution buffer and stored at −80°C.

Primers for ten human microsatellite loci (fluorescently labeled forward and unlabeled reverse; Amelogenin, TPOX, D3S1358, FGA, CSF1P0, D7S820, D8S1179, TH01, D13S317, D16S539; Table 1) [36] were used to amplify fragments of the extracted DNA. A multiplex PCR consisting of 12.5 uL Qiagen Multiplex PCR master mix (containing HotStarTaq DNA polymerase, Multiplex PCR Buffer with 6 mM MgCl_2_ and dNTP mix), 2.5 uL of an equal mixture of the aforementioned primers, 0–4 µL water, and 1–5 µL extracted DNA sample was created for each extracted cheek swab sample and engorged mosquito abdomen. The optimized cycling conditions were as follows: initial 95°C hold for 15 minutes; 35 cycles of 94°C for 30 seconds (denaturation), 60°C for 90 seconds (annealing), and 72°C for 60 seconds (extension); 60°C hold for 30 minutes; final hold at 4°C. PCR products were diluted 1∶10 and 1∶5–1∶15 for human cheek swabs and mosquito abdomens, respectively, and 0.5 uL of product was added to 0.5 uL LIZ-600 standard (Applied Biosystems) and 9 uL Hi-Di Formamide (Applied Biosystems) on 96 well plates for fragment analysis using the Hitachi 3130 XL genetic analyzer. Genotypes were interpreted using PeakScanner software (Applied Biosystems).

**Table 1:** Sequences for the 10 primers (forward and reverse) used in the microsatellite analysis.

Primer Name		Sequence
**TPOX**	**Forward (labeled)**	ACTGGCACAGAACAGGCACTTAGG
	**Reverse**	GGAGGAACTGGGAACCACACAGGTTA
**D3S1358**	**Forward (labeled)**	ATGAAATCAACAGAGGCTTGC
	**Reverse**	ACTGCAGTCCAATCTGGGT
**FGA**	**Forward (labeled)**	GGCTGCAGGGCATAACATTA
	**Reverse**	ATTCTATGACTTTGCGCTTCAGGA
**CSF1PO**	**Forward (labeled)**	ATTTCCTGTGTCAGACCCTGTT
	**Reverse**	CCGGAGGTAAAGGTGTCTTAAAGT
**D7S820**	**Forward (labeled)**	ATGTTGGTCAGGCTGACTATG
	**Reverse**	GATTCCACATTTATCCTCATTGAC
**D8S1179**	**Forward (labeled)**	GATTCCACATTTATCCTCATTGAC
	**Reverse**	ATGTTGGTCAGGCTGACTATG
**TH01**	**Forward (labeled)**	ATTCAAAGGGTATCTGGGCTCTGG
	**Reverse**	GTGGGCTGAAAAGCTCCCGATTAT
**VWA**	**Forward (labeled)**	GCCCTAGTGGATGATAAGAATAATCAGTATGTG
	**Reverse**	GGACAGATGATAAATACATAGGATGGATGG
**D13S317**	**Forward (labeled)**	GATTACAGAAGTCTGGGATGTGGAGGA
	**Reverse**	GGCAGCCCAAAAAGACAGA
**D16S539**	**Forward (labeled)**	GGGGGTCTAAGAGCTTGTAAAAAG
	**Reverse**	GTTTGTGTGTGCATCTGTAAGCATGTATC
**AMELOGENIN**	**Forward (labeled)**	ACCTCATCCTGGGCACCCTGG
	**Reverse**	AGGCTTGAGGCCAACCATCAG

#### Blood meal identification

Allelic profiles of blood meals were matched to those from cheek swabs using Mosquito Matcher 3.0 (blood meal identification software is available from the authors upon request). This program lists the identification codes of all individuals whose blood might have been in the abdomen, with percent match of cheek swab to blood meal and blood meal to cheek swab. Only those that matched >95% in both directions were considered complete matches. Due to the high percent match, abdomens that contained more than one blood meal from different individuals were not included in the analysis.

### Analysis

#### Statistical model

Under our model, during the week of each interview *k*, at each home *j*, every individual *i* that visits that home has a “biting suitability score,” *B_i|j,k_*, that depends in some way on their personal attributes: here, time spent at house *j*, *T_i|j,k_*, and body surface area, *S_i,k_*, although any other measurable attribute could also be considered. We define this score as the product of two functions, *t* and *a*, of the variables on which it depends; i.e.,
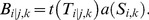
(2)While an individual's risk of being bitten at a particular house *j* will depend on his or her *B_i|j,k_*, it will also depend on the *B_l|j,k_* of each other person *l* that frequents house *j* during the week of interview *k*. Thus, we define the probability *P_i|j,k_* that a bite taken in house *j* during the week of interview *k* is taken on individual *i* as
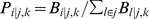
(3)Ultimately, these probabilities provide a link between data on which people received bites and various hypotheses about how individual characteristics contribute to who is bitten.

#### Hypotheses

We considered several individual level factors expected to influence the probability of a person being bitten by a female *Ae. aegypti*: body surface area (*S_i|j,k_*), age (*A_i|j,k_*), number of times entering the house (*E_i|j,k_*), weekly time in house (*T_i|j,k_*), and gender (*G_i_*). Each of these factors was tested individually and in every pairwise combination. Because we suspected that the relationship between biting and these factors could be saturating (e.g., after some time threshold, spending more time in the house does not increase the risk of a bite [9]), we considered a power functional relationship in addition to a linear function (Table 2).

**Table 2:** Models of biting suitability scores for (A) linear and (B) power based models, incorporating time in house (T), surface area (S) and the interaction of the two.

A) Linear Models	Null: 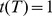 , 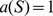
	T: 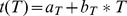 , 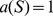
	S: 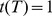 , 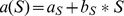
	TxS: 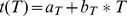 , 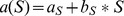
B) Power Models	Null: 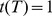 , 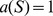
	T: 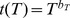 , 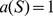
	S: 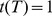 , 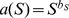
	TxS: 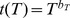 , 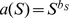

#### Likelihood evaluation and maximization

We assumed the recipients of bites were independent and the biting probabilities described by Eq. 3 were effectively constant. Thus, if *n_j,k_* of the bites that occurred in house *j* during study period *k* were received by the residents of house *j*, they would be distributed according to a multinomial distribution with the individual probabilities of being bitten given by Eq 3 with the denominator summing over only the residents of the home. If *J* is the set of all homes where at least one blood meal was matched to a member of the home, and *K* is the set of interview times (with the number of bites each individual *i* of each house *j* receives over interview time *k* denoted *x_i|j,k_*), the likelihood of the data given the *P_i|j,k_*'s is:
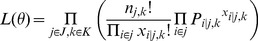
(4)The best-fit parameter values 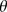 for each hypothesis were obtained by maximizing the likelihood of the data across all possible values of the parameters of each hypothesis. To facilitate interpretation of best-fit parameter values across the different hypotheses, we standardized them such that both the maximum *t* and the maximum *a* were 1. Doing so had no effect on their estimation.

#### Model comparison

The relative fit of nested models was assessed with likelihood ratio tests (LRT) and Akaike's Information Criterion (AIC).

## Results

### Data

#### Participants

**Figure 2:**
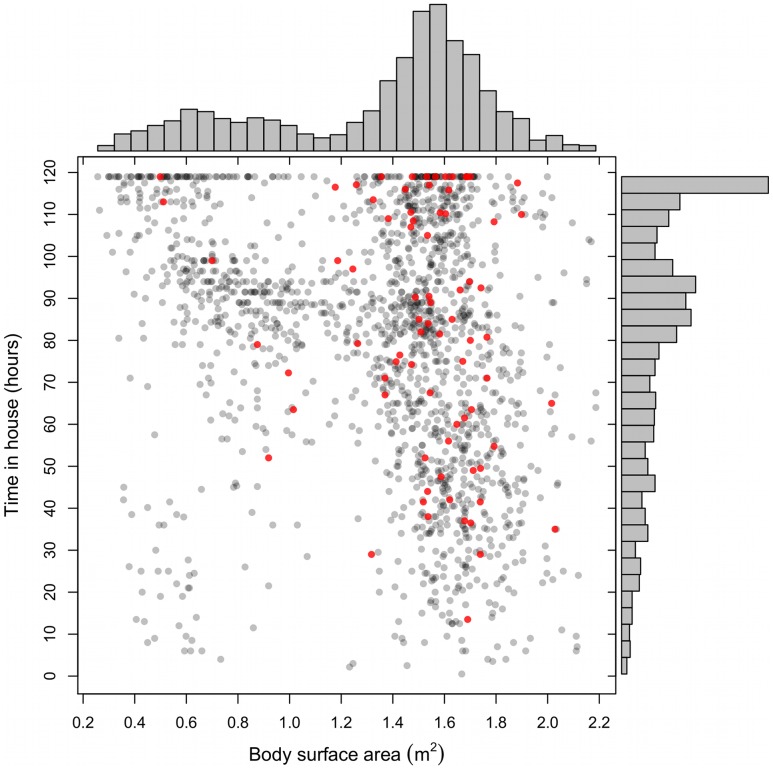
Joint distributions of reported total weekly time in a house and body surface area across all study participants measured on eight separate occasions. Red dots indicate individuals whose blood was identified in mosquitoes.

A total of 280 people from 19 households (8–29 per house, 53% male) participated in the study. Overall, 92% of all household residents participated, although there was variation among houses (58%–100%) due to changes in household composition over the 12-month study. The ages of participants ranged from 1 month to 75 years. Participant body surface area varied from 0.2 to 2.2 m^2^ (Figure 2), and increased predictably with age (data not shown).

#### Interviews and mosquito collections

1,647 activity interviews were conducted. More than one interview was obtained for 94% of the participants (N = 263; Figure S2). The majority of interviews (N = 1,178) were conducted on Saturday. Others were conducted throughout the week as participants' schedules permitted. Only 46 surveys were purely retrospective (conducted on Friday). Generally, younger (smaller) individuals reported spending more time in the house each week than adults (Figure 2). Between October 2009 and October 2010, 1,289 female *Ae. aegypti* were collected from the 19 households enrolled in the study.

#### DNA extraction and microsatellite analysis

Cheek swab samples from 275 of the 280 participants yielded complete DNA profiles. For the five participants for whom DNA did not amplify, new samples could not be obtained, because they were no longer residing in the city. Unique profiles were identified for all but four of the 275 participants, because two sets of identical twins were enrolled in this study. This presented no issues with identification, because no identified mosquito blood meals came from either set of twins. In total, 805 engorged and partially engorged blood meals from participating households were extracted. DNA amplification produced complete profiles for 110 samples. It is possible that some of the unamplified blood meals came from non-human hosts. Previous studies have shown, however, that *Ae. aegypti* feed predominantly on humans and, therefore, we did not run all samples against species-level primers [4], [5]. Prior to extracting DNA, we experienced storage and freezer malfunctions, which resulted in sample degradation. After repeated attempts we were unable to amplify DNA in most of the mosquitoes, which likely contributed to our ability to profile only 110 of the mosquito blood meals.

#### Blood meal identification

Unidentified human hosts were detected in 13% (N = 14) of the mosquito abdomens that yielded full DNA profiles. The blood in these samples could have come from a neighbor, visitor, field worker or possibly one of the five participants for whom DNA profiles were not obtained. The remaining 96 samples were matched to 68 study participants. Due to the degradation of DNA, we were not confident in using samples indicating multiple blood meals in our analyses.

### Analyses

**Figure 3:**
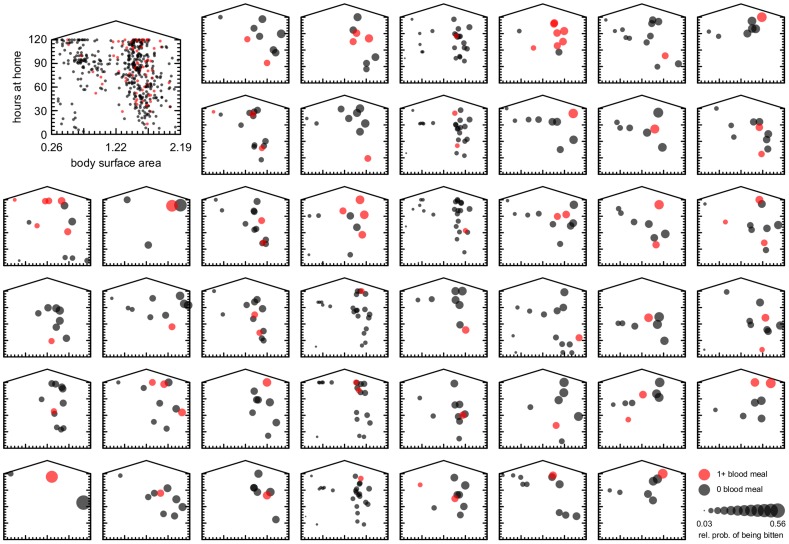
Joint distributions of reported total weekly time in house and body surface area in the 43 house-interview pairs in which there were positive blood meal identifications. Dot size is proportional to each individual's relative probability of being bitten (eq. 3), and red dots indicate individuals whose blood was identified in mosquitoes.

There was a high correlation between body surface area and age (until adulthood), and between the number of entrances and total weekly time in house. Additionally, the fitted relationship between biting score and either age, entrances, or both was weaker than those with surface area and time in house (age and gender were not significant predictors). Our primary analysis thus only includes time in house and surface area. The aggregated data on time in house and surface area (Figure 2) indicate that the majority of smaller individuals (children) spent more than half of their time in the home. There was no indication that smaller individuals (less than 1 m^2^ body surface area) that spent less than 50 hours in a week in their home were bitten by an engorged mosquito. Ultimately, however, whether an individual received a bite depended not only on their attributes, but also on the attributes of other residents in their house. In many of the houses in which blood meals were positively identified, larger people and those who spent more time at home tended to be the ones who were bitten (Figure 3).

**Figure 4:**
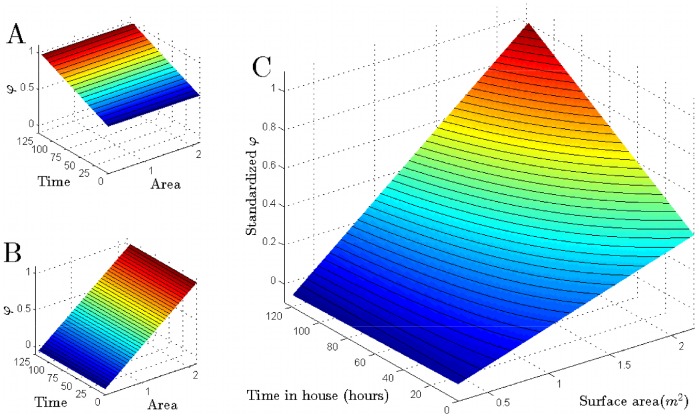
Fitted relationships between time in house, surface area and biting suitability using linear models. For each of the three linear model hypotheses described in Table 2 (A), the expected biting suitability score (*B_i_*) for an individual *i* with various combinations of time in house (*T_i_*) and surface area (*S_i_*) is plotted. In (A), an individual's biting score is independent of their surface area. In (B), an individual's biting score is independent of the amount of time they spend in the house. In (C), both factors influence biting score.

When weekly time-in-house alone was included in the model as a linear predictor of biting score, the fit was poor (Table 3; LRT: *p* = 0.247; Fig. 4A). The shallow slope of the fitted curve indicates that individuals who spent little time in a house did not have significantly lower biting scores than those who spent more time in the same house. Surface area, by contrast, was highly significant by itself (LRT: *p*<0.001; Figure 4B). Combining time in house and surface area improved the model's fit (Figure 4C; lower AIC), but this was not significantly better than the model with surface area by itself (LRT: *p* = 0.066; Table 3).

**Table 3:** Results of the linear models and model comparisons.

					Model Comparison
Model	mle	*LL*	d.f.	AIC	Null	T	S
**Null**	-	−168.54	0	337.08	-	-	-
**T**	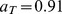 , 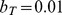	−167.87	1	337.74	NS	-	NS
**S**	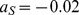 , 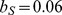	−160.61	1	323.22	***	†††	-
**T×S**	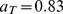 , 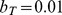	−158.92	2	321.84	***	***	+
	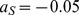 , 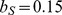						

Model comparison columns show significance of LRT or AIC tests comparing the model (in the far left column) with alternatives.

NS, non-significant;

+, p<0.10;

*** ,p<0.001;

††† , ΔAIC>10.

**Figure 5:**
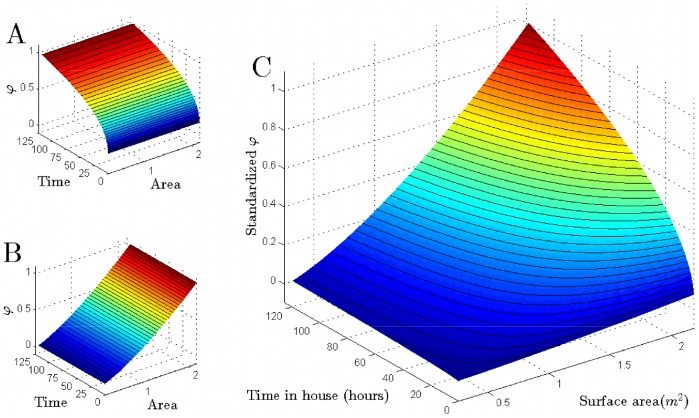
Fitted relationships between time in house, surface area and biting suitability using power models. For each of the three power model hypotheses described in Table 2 (B), the expected biting suitability score (*B_i_*) for an individual *i* with various combinations of time in house (*T_i_*) and surface area (*S_i_*) is plotted. In (A), an individual's biting score is independent of their surface area. In (B), an individual's biting score is independent of the amount of time they spend in the house. In (C), both factors influence biting score.

Models using power functions gave similar qualitative results to the linear models (Table 4), but somewhat different quantitative results (Figure 5). Weekly time-in-house was still a poor predictor by itself, and with the power functional form there was a sub-linear response (fitted power term 0.322). In other words, as time in house doubles, the biting score less than doubles. As with the linear models, a power function of surface area by itself also did a good job of explaining heterogeneous biting patterns in the data (Table 4; Figure 5B; *p*<0.001). In contrast to time in house, surface area had a super-linear relationship (fitted power term 1.4), indicating that incremental increases in surface area result in more than equivalent increases in biting score. Combining surface area with time in house again had the best AIC of all power models, and significantly improved model fit (p = 0.038; Table 4; Figure 5C). Biting probabilities predicted by the power model with time in house and surface area are shown in Figure 3 for each house in which a human source of a blood meal was positively identified.

**Table 4:** Results of the power models and model comparisons.

					Model comparison
Model	mle	*LL*	d.f.	AIC	Null	T	S
**Null**	-	−168.54	0	337.08	-	-	-
**T**	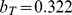	−167.434	1	336.87	NS	-	NS
**S**	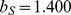	−160.796	1	323.59	***	†††	-
**T×S**	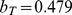	−158.643	2	321.286	***	***	*
	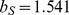						

NS, non-significant;

+ , p<0.10;

* ,p<0.05;

*** ,p<0.001;

††† , ΔAIC>10.

Neither linear nor power functions provided better fits to the data, both having similar optimal AIC scores (321.84 vs 321.286).

## Discussion

Understanding how female *Ae. aegypti* distribute their bites among human hosts is necessary to develop accurate models that ultimately assist in the design and implementation of more efficacious surveillance and disease control strategies. Our results indicate that, within a given household in Iquitos, *Ae. aegypti* more often bit larger people and those spending more time in the house, highlighting the importance of human movement behavior in determining individual risk of exposure to the viruses *Ae. aegypti* transmit. These factors predispose some individuals to receive more bites than others, with potentially important epidemiological effects. For instance, we expect the role of children in transmission to be less during the invasion of a new serotype, when immunologically naïve adults can become infected with, amplify, and transmit the virus. Under endemic transmission, however, infective bites are likely to fall on previously infected and thus immune adults, dampening transmission potential. Although future studies may elaborate on the determinants of heterogeneous biting, our results present a methodological advance in the analysis of DNA profiling data and empirical insight into the causal factors of *Ae. aegypti* biting and, by extension, DENV transmission.

Previous studies identified human body size as a potentially important predictor of who receives the most bites from anopheline mosquitoes [18]. Explanations include more surface area for biting, easier detectability due to increased CO_2_ production, a larger heat signature, reduced defensive behavior, and differences in host activity level [7], [26], [27], [28], [29]. Although our study design does not allow us to determine which of these or other mechanisms might explain the pattern we observed in Iquitos, the significance of our result across multiple models for body surface area in a house is consistent with the idea that mosquitoes are following cues (olfactory and/or visual) when selecting a host to feed upon. This effect of body surface area does appear, however, to be modulated somewhat by the amount of time that individuals spend at home. The significant increase of fit in the power model when incorporating both body surface area and time-in-house, and the marginally significant increase in the linear model (p-value = 0.066), are consistent with the hypothesis that people accumulate more bites at a location if they spend more time there. Our results indicate that this effect of total time-in-house is saturating and relatively weak, and other work is suggestive of an even weaker effect whereby frequency of visitation, but not duration, drives exposure to *Ae. aegypti* bites and infection risk [12]. To clarify what appears to be a nuanced effect of time-in-house on biting risk, we also considered models with more complex representations of time-in-house, but found them to be inconclusive given the available data. In combination, our results suggest that one's risk of being bitten is driven primarily by sensory cues that *Ae. aegypti* use to detect people. Future work with larger sample sizes and more detailed accounting of time-in-house and movement in and out of the house would help to further resolve the determinants of relative biting risk within a person's home.

As we and others have shown [13], [14], [15], [16], [17], [18], [19], [20], not all hosts have an equal probability of being bitten by mosquito vectors. The assumption of homogeneous biting has historically been used in calculations to determine how difficult an infectious disease is to control [21]. The most common measure of this, the basic reproductive number [37], is predicted to be higher in calculations based on models that allow for heterogeneous biting than in calculations based on models that assume homogeneous biting [22], [23], [24]. This indicates that controlling transmission could be more difficult than predicted by models that assume that all hosts have the same probability of being bitten. If, however, individuals who receive the most bites are identifiable, it may be possible to target interventions and more efficaciously control disease [20].

There were several limitations in our study regarding collection of adequate data for fitting our models. Due to technical issues associated with not being able to fingerprint all of the engorged mosquitoes we collected, we were limited in our ability to test alternative models defining biting risk. This included more complicated relationships between biting and the specific times at which participants were home. Although we had detailed time in house information for human household residents, we did not keep track of non-residents visiting the house or the risk of a resident being bitten at other places they visited during their daily activities [38]. Visitors might have influenced mosquito-biting decisions. The design of our study also precluded us from defining some individual attributes that might independently influence host attractiveness to mosquitoes, such as skin microflora [26].

We were, however, able to isolate important effects that influence how *Ae. aegypti* bites are distributed among its natural human hosts. Doing so required introducing a new statistical framework for assessing the contributions of different personal factors to one's relative risk of being bitten. Follow-up studies on *Ae. aegypti* or other household-biting mosquitoes should similarly account for the time people spend in a house and weight each individual's risk relative to other household residents. In particular, our results validate previous studies pointing to adults and/or larger people as the primary recipients of mosquito bites and underscore the importance of the time people spend at a location where mosquitoes bite. Moreover, our analyses reveal that the relationships between such factors can have nonlinear effects on an individual's risk, with time in house having a sub-linear effect and body surface area having a super-linear effect. More detailed understanding of these and other factors that contribute to an improved understanding of biting risk will be an important component of efforts to target interventions, such as vaccines for dengue virus that are currently under development.

## Supporting Information

Figure S1 Number of interviews conducted by day of the week.
(TIF)

Figure S2 Number of total interviews conducted on individuals.
(TIF)
